# Virulence Determinants and Antimicrobial Resistance Patterns of Vancomycin-resistant *Enterococcus faecium* Isolated from Different Sources in Southwest Iran

**Published:** 2018-02

**Authors:** Maniya ARSHADI, Mahmood MAHMOUDI, Moloud Sadat MOTAHAR, Saber SOLTANI, Mohammad Reza POURMAND

**Affiliations:** 1. Dept. of Pathobiology, School of Public Health, Tehran University of Medical Sciences, Tehran, Iran; 2. Dept. of Epidemiology and Biostatistics, School of Public Health, Tehran University of Medical Sciences, Tehran, Iran; 3. Dept. of Microbiology, School of Medicine, Ahwaz Jundishapur University of Medical Sciences, Ahwaz, Iran; 4. Urology Research Center, Tehran University of Medical Sciences, Tehran, Iran

**Keywords:** *Enterococcus faecium*, Vancomycin-resistant enterococci, Antibiotic resistance, Virulence determinants

## Abstract

**Background:**

This study aimed to investigate the incidence of antibiotic-resistance and virulence genes in vancomycin-resistant *Enterococcus faecium* isolated from different sources in southwest Iran from Mar to Sep 2015.

**Methods:**

Overall, 120 *E. faecium* isolates (80 VRE and 40 vancomycin-susceptible enterococci [VSE] isolates) were obtained from four hospitals. The resistance of the VRE isolates was determined by disk diffusion method. Multiplex PCR was performed to detect the virulence genes carried by the *E. faecium* isolates, namely, enterococcal surface protein (*esp*), hyaluronidase (*hyl*), and collagen-binding adhesin (*acm*).

**Results:**

All the VRE isolates exhibited multidrug resistance, with the rates of resistance to ampicillin, erythromycin, and ciprofloxacin reaching high levels. The isolates were least resistant to chloramphenicol and nitrofurantoin, but all of them were susceptible to linezolid. 46.6%, 20.8%, and 86.6% of the *E.* *faecium* isolates carried the *esp*, *hyl*, and *acm* genes, respectively.

**Conclusion:**

There is a significant difference between the prevalence of *esp* and *hyl* genes in the VRE and VSE isolates. In the VRE isolates, the high prevalence of multidrug resistance were found and the difference in the prevalence of *esp* among various sources was significant. The findings reflected a relationship between the prevalence of *esp* and *hyl* and resistance to certain antibiotics.

## Introduction

Enterococci, which exist in the gastrointestinal tract of humans, have been recognized as an important cause of healthcare-associated infections (1). These pathogens frequently cause urinary tract and wound infections, endocarditis, and bacteremia in hospitals (2). Enterococci display intrinsic and acquired resistance to many antimicrobial agents. For example, they are inherently resistant to cephalosporin, macrolides, clindamycin, trimethoprim-sulfamethoxazole, and low-level aminoglycoside. They have also acquired resistance to high-level aminoglycosides, vancomycin, ampicillin, and ciprofloxacin (3, 4).

Most enterococcal infections are caused by *Enterococcus faecalis* and *E. faecium*, but the majority of VRE infections are attributed to the latter (1). Nine types of glycopeptide resistance determinants (v*anA*, van*B*, van*C*, van*D*, *vanE*, *vanG*, v*anL*, *vanM*, and *vanN)* have been reported; among these, the most predominant glycopeptide resistance phenotype is *vanA*, followed by *vanB* (5). The acquisition of resistance genes by *E. faecium* presents a serious challenge to clinicians in treating enterococcal infections (6). Antimicrobial resistance can be transferred between enterococci isolates from different sources (7). Thus, effectively managing *E. faecium* infection necessitates identifying the resistance of isolates against different antibacterial drugs in every geographic region.

Virulence factors, along with antibiotic resistance, have been regarded as important determinants of the bacterial colonization, establishment, and persistence of vancomycin-resistant enterococci (VRE) in hospital settings (8). The presence of these factors provides bacteria the opportunity to adapt to hospital environments and acquire antibiotic-resistance genes. *E. faecium* carries multiple virulence factors, such as enterococcal surface protein (*esp*), hyaluronidase (*hyl*), and collagen-binding adhesin (*acm*) (9), whose expression is associated with colonization, host invasion, persistence, biofilm formation, and pathogenicity (10). The majority of hospital-associated strains of vancomycin-resistant *E. faecium* acquire virulence traits, including *esp*, *hyl,* and *acm*, thereby potentially increasing bacterial fitness in hospital settings (11). Accurate and reliable knowledge about various virulence factors and their functions is therefore essential in the design of VRE treatment.

The distribution of virulence genes was evaluated and compared in VRE and vancomycin-susceptible (VSE) *E. faecium*, but further studies should be carried out to clarify certain issues (12, 13). To date, limited data have been derived regarding the distribution of potential virulence genes among VRE *faecium* isolated from the different source in Iran and the most of the studies determined the prevalence of virulence genes in clinical isolates (14,15). There is no study in southwest Iran.

Accordingly, we investigated the association between virulence genes and antibiotic susceptibility profiles in clinical, intestine-colonizing, and environmental isolates of vancomycin-resistant *E.* *faecium* in southwest Iran.

## Materials and Methods

### Study design and sampling

The study was performed in four university hospitals (Abouzar, Imam Khomeini, Golestan, and Shafa) in Ahwaz City in southwest Iran from Mar to Sep 2015. The clinical and intestine-colonizing samples were collected from hospitalized patients, and the environmental samples were obtained from patients’ beds, ward sinks, tables, and medical equipment. Clinical samples including urine, wound, blood and catheter tips, were inoculated on blood agar medium (Merck). The plates were incubated overnight at 37 °C.

Ethical approval to conduct the study was granted by the Research Ethics Committee of the Tehran University of Medical Science, Tehran, Iran.

For intestine-colonizing and environmental sampling, each swab was moistened with sterile normal saline and used for sampling of rectal or environmental sources. Following sampling, each swab was inoculated on bile esculin Broth (Merck) and incubated overnight at 37 °C, and then they were subcultured on Blood agar medium. In this cross-sectional study, we examined 120 *E. faecium* isolates including 80 VRE isolates (35 clinical, 24 intestines colonizing, and 21 environmental) and 40 VSE isolates (16 clinical, 14 intestines colonizing, and 10 environmental).

### Species identification

Genus and species identification were performed by gram staining, catalase test, growth in the presence of 6.5% NaCl, bile-esculin test (Merck), and fermentation of arabinose, arginine, and sorbitol (Sigma Co) (16). The phenotypic identification was confirmed by PCR using species-specific primers (Table 1), and the genomic DNA of *Enterococcus* was extracted by boiling. PCR amplification involved initial denaturation at 95 °C for 4 min, followed by 35 cycles of denaturation at 94 °C for 30 sec, annealing at 54 °C for 1 min, extension at 72°C for 1 min, and a final extension at 72°C for 7 min (17). Vancomycin-resistant isolates were recognized by growth in bile-esculin agar containing 6 μgr/ml vancomycin (Sigma Aldrich, Germany).

**Table 1:** Primers used in this study

***Target Genes***	***Primer name***	***Sequence(5′–3′)***	***Size(bp)***	***References***
*ddl*	*ddl E.faecium-F* *ddl E.faecium-R*	TTGAGGCAGACCAGATTGACG TATGACAGCGACTCCGATTCC	658	(17)
*vanA*	*vanA-F* *vanA-R*	CATGAATAGAATAAAAGTTGCAATA CCCCTTTAACGCTAATACGATCAA	1030	(17)
*vanB*	*vanB-F* *vanB-R*	GTGACAAACCGGAGGCGAGGA CCGCCATCCTCCTGCAAAAAA	433	(17)
*esp*	*esp-F* *esp-R*	AGATTTCATCTTTGATTCTTGG AATTGATTCTTTAGCATCTGG	510	(18)
*hyl*	*hyl-F* *hyl-R*	ACAGAAGAGCTGCAGGAAATG GACTGACGTCCAAGTTTCCAA	276	(18)
*acm*	*acm-F* *acm-R*	GGCCAGAAACGTAACCGATA AACCAGAAGCTGGCTTTGTC	135	(8)

F, Forward (upstream) primer; R, Reverse (downstream) primer

### Antimicrobial susceptibility test

We tested the antimicrobial susceptibility of the vancomycin-resistant *E. faecium* isolates to eight agents, namely, ciprofloxacin (5 μg), nitrofurantoin (300 μg), ampicillin (10 μg), linezolid (30 μg), teicoplanin (30 μg), gentamycin (120 μg), erythromycin (15 μg), and chloramphenicol (30 μg). The test was conducted by disk diffusion method in accordance with the recommendations of the Clinical and Laboratory Standard Institute Guideline, and *E. faecalis* ATCC 29212 was used as the quality control strain. The antibiotic disks used in the research were supplied by MAST Laboratories Ltd. (MAST Diagnostics, UK). The Minimum Inhibitory Concentrations (MICs) of vancomycin and teicoplanin were determined by E-test (Liofilchem, Italy).

Multiplex PCR assay was performed to detect vancomycin-resistance genes (*vanA* and *vanB*), as previously described (17).

### Detection of virulence genes

Three major virulence genes *(esp*, *hyl*, *acm*) in the clinical, intestine-colonizing, and environmental VRE and VSE isolates were detected by multiplex PCR using the primers listed in Table 1. PCR amplification was carried out using amplification parameter as follows: initial denaturation at 94 °C for 5 min, followed by 30 cycles of denaturation at 94°C for 1 min, annealing at 54 °C for 1 min, extension at 72 °C for 1 min, an a final extension at 72 °C for 10 min (18). The PCR products were analyzed by 1% agarose gel electrophoresis (SinaClon, Iran).

### Statistical analysis

Data were statistically analyzed using chi-square and Fisher’s exact tests, and significant difference (*P*<0.05) was determined by SPSS software (ver. 22, Chicago, IL, USA).

## Results

Among the 120 *E. faecium* isolates, 80 (67%) and 40 (33%) were VRE and VSE isolates, respectively. Among the clinical isolates, the most common specimen was urine (62.7%, n=32), followed by wound (25.5%, n=13). The results of the PCR assay for the *ddl faecium* gene confirmed the phenotypic identification of the *E. faecium* isolates. All the VRE isolates harbored the *vanA* gene.

### Antibiotic resistance patterns

The 80 VRE isolates were tested for susceptibility to eight antimicrobial agents by disk diffusion method. The comparison results on antibiotic susceptibilities are shown in Table 2.

**Table 2:** Resistance patterns of VRE isolates to different antibiotics

***Antimicrobial agents***	***Clinical isolates N=35***	***Colonizing isolates N=24***	***Environmental isolates N=21***	***Total N=80***	***Test***	***P-value***
	***Resistance N (%)***	***Resistance N (%)***	***Resistance N (%)***	***Resistance N(%)***		
Ampicillin	32(91.4)	23(96.8)	19(91.3)	74 (92.5)	Fisher’s exact test	0.759
Teicoplanin	35(100)	24(100)	21(100)	80 (100)	--	--
Gentamycin	21(60)	20(83.5)	19(91.5)	60 (80)	Fisher’s exact test	0.023
Ciprofloxacin	33(97)	24(100)	20(95.5)	77(96.2)	Fisher’s exact test	0.995
Chloramphenicol	9(25.7)	10(41.6)	7(35)	26(32.5)	Chi-square test	0.436
Erythromycin	35(100)	21(91.6)	21(100)	77(96.2)	Fisher’s exact test	0.376
Linezolid	0	0	0	0	-	-
Nitrofurantoin	13(37)	8(33)	8(40)	29(38)	Chi-square test	0.936

All the VRE isolates presented multidrug resistance, with the rates of resistance to ampicillin (92.5%), teicoplanin (100%), ciprofloxacin (96.2%), and erythromycin (96.2%) being of high levels. The rate of resistance to gentamycin was higher in the environmental (91.5%) and intestine-colonizing isolates (81.5%) than in the clinical isolates (60%) (*P*<0.05). All the VRE isolates were susceptible to linezolid. The isolates exhibited the least resistance to chloramphenicol (32.5%) and nitrofurantoin (38%). Moreover, all the VRE isolates exhibited high MICs to vancomycin (64 to >256 μg/ml) and teicoplanin (16 to 256 μg/ml).

### Prevalence of virulence genes in VRE and VSE isolates

In terms of gene encoding for potential virulence factors, 46.6% (n=39), 20.8% (n=18), and 86.6% (n= 104) of the 120 *E. faecium* strains were positive for *esp*, *hyl*, and *acm*, respectively. The distribution of these virulence genes among the clinical, intestine-colonizing, and environmental VRE and VSE isolates is shown in Table 3 and 4.

**Table 3:** Distribution of virulence genes among VRE isolates from various sources

***Total*** ***N=80 (%)***	***Environmental isolates N=21 (%)***	***Intestine-colonizing isolates N=24 (%)***	***Clinical isolates N=35 (%)***	***Virulence genes***
46(57.5)	15(71.4)	7(29.1)	24(68.5)	*esp*
23(28.7)	9(42.8)	6(25)	8(22.8)	*hyl*
71(88.7)	20(95.2)	21(87.5)	30(85.7)	*acm*

**Table 4:** Distribution of virulence genes among VSE isolates from various sources

***Total N=40 (%)***	***Environmental isolates N=10 (%)***	***Intestine-colonizing N=14 (%)***	***Clinical isolates N=16 (%)***	***Virulence genes***
10(25)	1(10)	4(28.5)	5(31.2)	*esp*
2(5)	0(0)	1(7.1)	1(6.2)	*hyl*
33(82.5)	9(90)	10 (71.4)	14 (87.5)	*acm*

The *esp* gene was found in 57.5% (n=46) of the VRE isolates and 25% (n=10) of the VSE isolates. A significant difference in *esp* prevalence was found between the VRE and VSE isolates (*P*=0.001).

The *hyl* gene was found in 28.7% (n=23) of the VRE isolates but in only 5% (n=2) of the VSE isolates. A significant difference (*P*=0.003) in *hyl* prevalence was found between the VRE and VSE isolates. The *acm* gene was found in 88.7% (n=71) of the VRE isolates and 82.5% (n=33) of the VSE isolates. No significant difference in *acm* prevalence was found between the isolates (*P*=0.6). A significant difference in the prevalence of *esp* was found among the VRE isolates (*P*=0.003). No significant differences in *hyl* and *acm* positivity rates (*P*=0.2 and *P*=1, respectively) were found among the clinical, intestine-colonizing, and environmental VRE isolates.

## Discussion

The prevalence of antimicrobial-resistant microorganisms in various healthcare settings has increased dramatically in the last decade. The isolation of multiple drugs resistant Enterococci (MDR) has become worrisome worldwide. Moreover, enterococcus in food of animal origin, municipal and hospital sewage treatment plants (STPs), which could persist in the ecosystem for a long time, is under intensive investigations, especially when these resistant pathogens could spread through these sources to human community and hospital wards (19–21).

The spread of VRE in hospitals is a serious threat to patient care systems because these bacteria are not only resistant to vancomycin but also intrinsically and acquisitionally resistant to a wide range of antibiotics. They can also serve as a potential reservoir of resistance genes and can transfer to sensitive species of enterococci and other strains of bacteria, including MRSA (3). The presence of virulence determinants in enterococci isolates is very important for colonization and the progress and persistence of infections in hospitals. A necessary requirement, then, is precisely to identify virulence markers to determine the pathogenicity and severity of enterococcal disease (22).

In this study, 80 VRE and 40 VSE isolates collected from various clinical, intestine-colonizing, and environmental sources were evaluated for their antibiotic resistance patterns and the presence of *esp*, *hyl*, and *acm* genes. All the VRE isolates carried the *vanA* gene and exhibited high vancomycin and teicoplanin MICs. This finding is consistent with the results of the majority of researches in Iran (23, 24). All the VRE isolates showed resistance to multiple drugs, with high resistance observed particularly for ampicillin (92.5%), ciprofloxacin (96%), and erythromycin (96.2%). This result also aligns with the findings of other studies (23–26). In all these cases, no significant difference in resistance was found among the clinical, intestine-colonizing, and environmental isolates (*P*>0.05). In Iran, high levels of resistance to the drugs may be due to self-medication for the treatment of various infections, the prescription of drugs by physicians not reviewed the results of susceptibility tests, and the widespread use of drug derivatives, especially azithromycin. In this study, 80% of the VRE strains showed resistance to gentamicin, with the highest level observed in the environmental (91.5%) and intestine-colonizing (84%) isolates. The lowest level of resistance was observed in the clinical isolates (60%). The previous study in Tehran has reported a relatively high level of resistance to gentamicin among clinical isolates (23). We found no published reports on resistance level in our study region, but the differences in results may be attributed to the differences in the treatment policies of various regions. 

Such policies are designed based on regional resistance patterns. In our study, we observed a relatively low level of resistance to chloramphenicol (32%), which may be due to its limited clinical use in Iran. All the isolates were resistant to teicoplanin, consistent with the resistance genetic pattern of *vanA*. Of all the studied VRE isolates, 36.2% were resistant to nitrofurantoin, but resistance level did not significantly differ among the various isolate sources. All the VRE isolates exhibited linezolid sensitivity, which may have stemmed from the fact that this drug is not used in the studied region.

Among the *E. faecium* isolates, the most frequently identified virulence gene was *acm* (86.6%), followed by *esp* (46.6%) and *hyl* (20.8%). Enterococcal surface protein, coded by chromosomic *esp*, plays an important role in initial attachment of bacteria in the urinary tract, and the formation of biofilm (15). The results of this study showed that 46.6% of the *E.* *faecium* isolates carried the *esp* gene. This is consistent with the findings of another study (1), who reported the presence of this gene in 45.7% of *E. faecium* clinical isolates in France. Our findings contrast with other investigators (8,27), who reported that the incidence levels of *esp* in *E*. *faecium* isolates were 70.9% and 4.3%, respectively. In our study, the level of *esp* incidence in the VRE isolates was 57.5%, which aligns with the incidence found by other researcher (44%) (28). The levels observed in our study were lower than those found by other studies in Iran (71.5%) (15) and China (90%) (10). As indicated by our results, the incidence of *esp* in the VRE isolates was significantly higher than that in the VSE isolates (*P*<0.05). This finding is consistent with the finding of other investigators (29, 30). Moreover, the number of positive *esp* genes found in the VRE isolates obtained from the clinical samples were significantly higher than those found in the VRE isolates collected from the intestine-colonizing sources, suggesting the important role of *esp* in enterococcal pathogenesis. The *hyl* gene in the *E.* *faecium* encodes hyaluronidase; the presence of this gene is believed to facilitate intestinal colonization and invasion by bacteria (18). Various reports have been published regarding the presence of *hyl* in infectious and colonized isolates. In the current work, the percentage of positive *hyl* in the *E.* *faecium* isolates was 20.8%, which is consistent with the results of Strateva’s report of 24.2% gene frequency in Bulgaria (27). In the present study, *hyl* was found in 28.7% of the VRE isolates, which aligns with the findings of other researchers (10, 18, 31), who reported that the percentages of *hyl* in VRE isolates were 27.5%, 17%, and 27%, respectively. These levels, however, were lower than that found (52.2%) (1). In a study in Iran (32), none of the *E.* *faecium* isolates carried the *hyl* gene, but this result may have been due to the author’s use of water as the source of the studied samples. Generally, a comparison of the results of studies conducted on various samples in different parts of the world shows contradictory results regarding *hyl* incidence. Such contradiction may be ascribed to the diversity of studied communities and differences in sample sources. A significant difference in *hyl* incidence was found between the VRE and VSE isolates (*P*<0.05), consistent with the findings of another investigator (29). The incidence of *esp* and *hyl* virulence genes was higher in the VRE isolates than in the VSE isolates, suggesting the presence of nosocomial *E.* *faecium* strains. The *esp* and *hyl* genes are part of a pathogenicity island, and their coexistence with ampicillin and ciprofloxacin-resistance genes can act as a marker for bacteria outbreak and play a role in the spread of epidemic VRE isolates in hospital environments. Additionally, no significant difference in *hyl* incidence (*P*=0.2) was found among the VRE isolates from different sources. This finding is consistent with the results of another investigator (29). However, some studies have shown higher incidences of *hyl* in clinical VRE isolates than in colonizing VRE isolates (33).

The exact role of *acm* in the pathogenesis of *E.* *faecium* infection is unknown, but some studies have introduced it as a functional gene, whose product is one of microbial surface components recognizing adhesive matrix molecules (34). In the present research, a high level of *acm* incidence (86.6%) was observed in the *E.* *faecium* isolates, consistent with the results of other studies (32,35), who reported incidence levels of 84.7% and 100%, respectively. In contrast to studies that reported a higher spread of *acm* locus genes in clinical isolates of multidrug-resistant *E.* *faecium* isolates (36), the current research found no significant difference among the VRE isolates from various sources or between resistant and sensitive *E. faecium* isolates.

## Conclusion

This study found a high prevalence of multidrug resistance in VRE isolates taken from different sources. Such prevalence is a serious challenge to the treatment of enterococcal infection, especially in hospitalized patients. The findings also reflected a relationship between the prevalence of *esp* and *hyl* in the VRE strains and ampicillin and ciprofloxacin resistance that resembles the features hospital isolates of VRE *faecium*. Further studies are needed to definitively determine the association between enterococcal virulence and antibiotic resistance.

## Ethical considerations

Ethical issues (Including plagiarism, Informed Consent, misconduct, data fabrication and/or falsification, double publication and/or submission, redundancy, etc.) have been completely observed by the authors.
